# ACE2 Expression and Clinical Biomarkers in COVID‐19: Associations With Disease Severity

**DOI:** 10.1155/pm/1013605

**Published:** 2026-06-10

**Authors:** Kazhin Rahim Ali Saeed, Paywast Jamal Jalal

**Affiliations:** ^1^ Biology Department, College of Science, University of Sulaimani, Sulaymaniyah, Kurdistan Region, Iraq, univsul.edu.iq

**Keywords:** angiotensin-converting Enzyme 2, disease severity, gene expression, qRT-PCR, SARS-CoV-2

## Abstract

**Background:**

The COVID‐19 pandemic, caused by SARS‐CoV‐2, has led to significant global mortality and morbidity. Angiotensin‐converting Enzyme 2 (ACE2) has been identified as the primary receptor for viral entry into host cells and is believed to play a critical role in disease progression and severity. While ACE2 expression has been studied across various populations, data remains limited for underrepresented ethnic groups, such as the Kurdish population.

**Methods:**

A prospective pilot study was conducted with 45 participants, divided equally into three groups (*n* = 15 each): mild cases, severe cases, and healthy controls. Whole blood samples were collected to assess hematological and biochemical parameters. ACE2 mRNA expression was analyzed using quantitative real‐time PCR (qRT‐PCR). Group comparisons were performed using one‐way ANOVA for *Δ* *C* *q* values and nonparametric tests for nonnormally distributed variables.

**Results:**

ACE2 expression was significantly upregulated in patients with severe COVID‐19, as indicated by lower *Δ* *C* *q* values compared to controls (mean difference = 0.568; adjusted *p* = 0.032). No significant difference was observed between mild cases and controls (*p* = 0.138). Furthermore, severe disease was characterized by markedly increased inflammatory markers (CRP, ESR, and ferritin) and hepatic enzymes (AST/GOT and ALP), alongside a decrease in lymphocyte counts. Also, CRP, ESR, and GPT were identified as the most significant predictors of disease severity in a machine learning analysis using XGBoost with SHAP interpretation. Demographic analysis revealed a significant age difference between controls and patients; however, no difference was observed between mild and severe cases. The sex distribution was comparable across all study groups.

**Conclusion:**

Reduced lymphocyte counts and systemic inflammatory responses, specifically elevated CRP, ESR, and ferritin levels, were closely associated with disease severity in COVID‐19. Although ACE2 expression results provide preliminary evidence that the Kurdish cohort′s ACE2 mRNA expression is elevated in cases of severe COVID‐19, its contribution to disease classification was minimal, indicating that multiple inflammatory and RAAS‐related mechanisms, rather than ACE2 expression alone, drive disease progression.

## 1. Introduction

SARS‐CoV‐2 is the causative agent of COVID‐19, a highly contagious viral disease that first emerged in Wuhan, China, in December 2019 [1]. Since its onset, the COVID‐19 pandemic has triggered an unprecedented global health crisis; as of mid‐2025, there have been over 778 million confirmed cases and more than 7.1 million recorded deaths [2, 3]. While many individuals experience mild or asymptomatic illness, others progress to severe disease characterized by respiratory failure, multiorgan dysfunction, and death [4]. A critical determinant of SARS‐CoV‐2 infectivity is its reliance on angiotensin‐converting Enzyme 2 (ACE2) as the primary receptor for cellular entry. ACE2 is a Type I transmembrane protein widely expressed in alveolar lung cells, the heart, kidneys, gastrointestinal tract, liver, brain, and testicular tissues [5–7]. It shares 42% homology with the angiotensin‐converting enzyme (ACE) [8], is located on Chromosome Xp22, and consists of 17 introns and 18 exons [9, 10]. Beyond serving as a viral entry point, ACE2 plays an essential role in the renin–angiotensin system (RAS). It maintains physiological homeostasis by converting circulating Angiotensin II (Ang II) into Angiotensin 1–7 (Ang 1–7)—a peptide with vasodilatory and anti‐inflammatory effects that counterbalances the proinflammatory actions of ACE and Ang II [11, 12]. However, SARS‐CoV‐2 hijacks this enzyme by binding its spike (S) protein to the ACE2 receptor to gain entry into host cells [5, 13]. This process is further facilitated by host proteases, most notably the transmembrane serine Protease 2 (TMPRSS2), which primes the S protein to initiate infection [5].

Consequently, SARS‐CoV‐2 infection disrupts ACE2 expression and activity, which may contribute to systemic organ damage and increased disease severity [14]. Beyond pulmonary involvement, ACE2 is highly expressed in cholangiocytes in the liver, where it contributes to maintaining local RAS balance [15]. Binding of SARS‐CoV‐2 to ACE2 promotes dysregulation of hepatic RAS signaling, favoring Ang II–mediated pathways that increase AT1 receptor‐driven inflammation and fibrotic responses by releasing Inflammatory cytokines such as TNF‐*α* and IL‐6 [7]. Hepatocyte damage in COVID‐19 is therefore produced by oxidative, fibrotic, and apoptotic pathways that are activated by this RAS imbalance [7].

Similarly, ACE2 expression in neural tissues has been implicated in SARS‐CoV‐2, where viral interaction may induce the immune system through cytokine storms, leading to neuroinflammation, microglial activation, neuronal death, and amyloid‐*β* buildup, all of which contribute to Alzheimer′s disease progression [6]. Collectively, these extrapulmonary effects highlight the systemic relevance of ACE2 beyond viral entry and support its investigation as a biomarker of disease severity. However, the regulation of ACE2 expression during infection is complex. Some studies suggest downregulation due to viral internalization, while others report upregulation of ACE2 mRNA as a compensatory response (Gaber et al.). Recent findings have also introduced the concept of an interferon‐inducible truncated ACE2 isoform (dACE2), further complicating the interpretation of mRNA expression patterns [16].

Given the pivotal role of ACE2 in infection, its genetic polymorphism has drawn increasing attention. This gene is considered polymorphic, and numerous authors have hypothesized that single‐nucleotide polymorphisms (SNPs) in the ACE2 (Xp22.2) gene may affect gene expression and the binding affinity of SARS‐CoV‐2, thereby influencing the severity of COVID‐19 and susceptibility to SARS‐CoV‐2 infection [17, 18]. Furthermore, variations in SNPs produce differences in mRNA expression levels of the ACE2 gene among humans [19]. The varying individual vulnerability to SARS‐CoV‐2 infection and the various clinical outcomes can be explained by examining the ethnic variations in these polymorphisms [20]. Furthermore, research has identified sex‐specific SNPs associated with variations in disease severity. Males generally experience more severe illness than females, a discrepancy potentially driven by hormonal factors as well as the specific chromosomal location and expression patterns of the ACE2 gene [21]. Additionally, while some reports suggest that ACE2 mRNA expression levels may shift with age, conclusive evidence for this trend remains elusive [22]. Collectively, these findings have prompted further investigation into host genetic factors as key determinants of COVID‐19 severity.

In light of these findings, we hypothesized that differences in ACE2 gene expression are associated with clinical biomarkers and disease progression in patients with COVID‐19. These associations are modulated by age and sex. To investigate this, the mRNA expression of the ACE2 gene was assessed in peripheral blood rather than in tissue samples, since tissue biopsies are invasive and ethically impractical, especially in COVID‐19 patients. In contrast, blood sampling is a less invasive and safer method of evaluating systemic immunological and molecular responses. The evaluation was conducted among healthy individuals and COVID‐19 patients within a targeted cohort in Sulaymaniyah City, including both mild and severe cases. The study integrated demographic and clinical data through the use of molecular, statistical, and bioinformatic techniques. This multidimensional approach seeks to enhance our understanding of ACE2 regulation during COVID‐19 infection and provide population‐specific insights to the global body of literature. Due to limited research infrastructure and genomic resources in the region, little is known about ACE2 polymorphisms and their impact on ACE2 expression in the Kurdish population. The scarcity of large population datasets, limited access to advanced bioinformatic support, and the limitations of high‐throughput sequencing technology underscore the need for comprehensive molecular and genomic investigations in this field.

## 2. Materials and Methods

### 2.1. Study Population

According to a standardized questionnaire administered at the time of sample collection, individuals with known comorbidities or those taking medications that might influence ACE2 expression, such as ACE inhibitors or angiotensin receptor blockers (ARBs), were excluded. Accordingly, this prospective pilot study initially included 94 samples for RNA extraction and biomarker analysis. From this pool, 45 samples were specifically selected for ACE2 expression analysis to satisfy the stringent RNA quality requirements necessary for reliable RT‐qPCR. Selection was based on spectrophotometric evaluation and gel electrophoresis results, which assessed RNA concentration, purity (A260/A280 ratio), and overall structural integrity. Samples with low RNA concentration, poor purity, or evidence of degradation were excluded, likely due to RNA instability associated with long‐term storage during the 2020–2021 period. All patients were diagnosed with SARS‐CoV‐2 infection at Shorsh Hospital, with disease severity determined according to WHO classifications [23]. The inclusion period spanned 2020 and 2021, during the first wave of the pandemic; consequently, all patients were experiencing their first infection and had not been immunized or vaccinated.

Patients were categorized into two clinical groups based on the severity of their manifestations:

• Mild cases: Defined as individuals without hypoxia or radiological signs of pneumonia, presenting only with mild symptoms (e.g., fever and cough) and an oxygen saturation (SpO_2_) of 90% or higher.
• Severe cases: Defined as those requiring admission to the intensive care unit (ICU) due to severe hypoxia (SpO_2_ < 90%), following the diagnostic criteria of the National Health Commission of China and supported by WHO classifications [23, 24].

The control group consisted of healthy individuals who tested negative for COVID‐19 via RT‐PCR and were asymptomatic. These individuals also tested negative for other viral markers, including HBsAg, HBcAg, HCV, and HIV, and exhibited normal hematological and biochemical profiles. Note: Blood samples were collected at a single time point during the acute stage of infection. Severe cases were sampled shortly after admission to the ICU, whereas mild cases were sampled during the outpatient evaluation following confirmed diagnosis.

### 2.2. Sample Collection

Seven milliliters of venous blood was drawn from each participant via venipuncture using disposable syringes. From this volume, 2.5 mL was collected in EDTA tubes for complete blood count (CBC) and erythrocyte sedimentation rate (ESR) testing; these samples were subsequently stored at −80°C for future molecular analysis. The remaining 4.5 mL was collected in clot activator gel tubes, allowed to clot at room temperature, and centrifuged at 3500 RPM for 10 min. The resulting serum was then separated and utilized for biochemical assays.

### 2.3. Ethical Approval

Ethical approval for this study (Code: 346) was granted by the Ethics Committee of the College of Medicine at the University of Sulaimani, Sulaymaniyah, on October 23, 2024.

This study utilized preserved blood samples collected during previous COVID‐19‐related clinical and research activities. Most of the participants provided written informed consent at the time of sample collection. In some severe cases requiring ICU admission, verbal informed consent was obtained based on the patients′ critical clinical condition and in accordance with institutional ethical regulations and ethics committee approval, as written consent could not be obtained.

### 2.4. Hematological and Biochemical Analysis

All biochemical parameters—including aspartate aminotransferase (GOT/AST), alanine aminotransferase (GPT/ALT), alkaline phosphatase (ALP), total serum bilirubin (TSB), C‐reactive protein (CRP), and serum ferritin—were measured using a Cobas c311 analyzer (Roche Diagnostics, Switzerland) following standard protocols. CBCs and differentials were performed using a Swelab Alfa Plus automated three‐part differential hematology analyzer (Boule Medical AB, Stockholm, Sweden). The ESR (reference range: 0–20 mm/h) was determined using the Westergren method.

### 2.5. RNA Extraction

Total RNA was extracted from whole blood samples using the QIAamp RNA Blood Mini Kit (Qiagen, Germany; Cat. No. 52304) in strict accordance with the manufacturer′s instructions. The procedure began with the separation of buffy coat, which was subsequently mixed with erythrocyte lysis buffer (Buffer EL) at a 1:5 ratio and incubated on ice to lyse red blood cells. Following centrifugation, the leukocyte pellet was resuspended and washed to ensure complete removal of erythrocytes. The leukocyte lysate was prepared by using Buffer RLT supplemented with *β*‐mercaptoethanol, followed by homogenization of the lysate on a QIAshredder spin column.

Ethanol was then added at approximately a 1:1 ratio to facilitate RNA binding, and the mixture was transferred to a QIAamp silica spin column. Subsequently, the column was washed sequentially with Buffer RW1 and Buffer RPE to remove contaminants. RNA was eluted with RNase‐free water and stored at −80°C until RT‐qPCR analysis, which was performed shortly after extraction.

The purity and concentration of the extracted RNA samples were assessed using a NanoDrop spectrophotometer (EzDrop 1000, Blue‐Ray Biotech), and only samples demonstrating high integrity and optimal purity were included for downstream molecular applications, such as expression analysis, as described previously. Selected RNA samples were stratified into three clinical groups (mild = 15, severe = 15, and healthy control = 15), as shown in Table 1.

**Table 1:** Demographic distribution of study participants by group severity, gender, and age.

Group	Female (*n*)	Male (*n*)	Age range (years)	Total (*n*)
Control	4	11	18–53	15
Mild	10	5	32–90	15
Severe	6	9	30–80	15
Total	20	25	18–90	45

*Note:* Forty‐five participants (20 females and 25 males) were used.

### 2.6. ACE2 Real‐Time PCR

After RNA extraction, primers for ACE2 mRNA variants were designed and verified using primer design software to minimize nontarget and secondary structures. The details are presented in Table 2.

**Table 2:** Primer sequences and characteristics used for ACE2 gene expression analysis by qRT‐PCR.

Primer name	Sequence (5 ^′^ → 3 ^′^)	Length (bp)	%GC	Tm (°C)	Product size (bp)
ACE2‐Human‐F	GGAGTTGTGATGGGAGTGATAG	22	50%	53°C	117 bp
ACE2‐Human‐R	ATCGATGGAGGCATAAGGATTT	22	49.9%	53°C	

Primers were synthesized by Sigma‐Aldrich/Merck KGaA, Germany. Primers′ specificity was evaluated in silico using NCBI Primer‐BLAST against the human reference genome. Stock solutions of each primer were diluted in nuclease‐free double‐distilled water (ddH_2_O) to a working concentration of 10 pmol/*μ*L.

The mRNA expression of ACE2 was quantified using one‐step RT‐qPCR with the qPCRBIO SYGreen One‐Step Mix (PCR Biosystems, United Kingdom). The reaction components are summarized in Table 3. Quantitative real‐time PCR (qRT‐PCR) was performed using the CFX96 Real‐Time PCR Detection System (Bio‐Rad Laboratories, United States).

**Table 3:** RT‐qPCR reaction composition.

Component	Volume (per reaction)	Final concentration
2× qPCRBIO SYGreen Mix	10.0 *μ*L	1×
Forward primer (10 *μ*M)	0.8 *μ*L	400 nM
Reverse primer (10 *μ*M)	0.8 *μ*L	400 nM
RTase Go enzyme	1.0 *μ*L	—
RNA template	X *μ*L	Normalized concentration
Nuclease‐free ddH_2_O	Up to 20 *μ*L	—

*Note:* X is adjusted per sample to ensure template consistency with ddH_2_O, making up the remaining volume.

The qRT‐PCR conditions were as follows: an initial reverse transcription step at 50°C for 10 min, followed by initial denaturation at 95°C for 2 min. This was followed by 40 cycles of denaturation at 95°C for 30 s and annealing/extension at 53°C for 30 s, during which fluorescence data (SYBR Green) was acquired. Amplification specificity was subsequently confirmed via melt curve analysis. Glyceraldehyde‐3‐phosphate dehydrogenase (GAPDH) was used as an internal reference gene to normalize gene expression across all samples. Reactions were performed in triplicate, and a no‐template control (NTC) was included in every run by replacing the RNA template with ddH_2_O to ensure the absence of contamination. The results were then subjected to a 2^−*Δ* *Δ* *C* *q*^ calculation to evaluate the relative fold expression of the ACE2 gene target.

## 3. Statistical and Bioinformatic Analysis of Biological and Molecular Parameters

For all analyses, *α* = 0.05 was used as the a priori level of statistical significance. GraphPad Prism (Version 9.0) was utilized for the analysis of all hematological and biochemical data. The normality of the data distribution was assessed using the Shapiro–Wilk test. Because most variables were not normally distributed, nonparametric analyses were employed. To maintain data quality, outliers identified via Prism′s automatic outlier detection were excluded from the analysis.

Differences among the three study groups (control, mild, and severe COVID‐19 patients) were evaluated using the Kruskal–Wallis test, followed by Dunn′s multiple comparisons post hoc test. Results are expressed as mean ± standard deviation (SD), and statistical significance was defined as *p* < 0.05.

Before outcome analysis, baseline demographic comparability was evaluated. The Kruskal–Wallis test with Dunn′s post hoc comparisons was used to assess age distributions across groups, while the chi‐square test was used to examine sex distribution.

To account for the variation in RNA input and reverse transcription efficiency, relative ACE2 gene expression was normalized to GAPDH, an endogenous reference gene. The *Δ* *C* *q* values for each sample were calculated by subtracting the GAPDH *C* *q* from the ACE2 *C* *q* (*Δ* *C* *q* = *C* *q* (ACE2) − *C* *q* (GAPDH)).

The control, mild, and severe groups were compared using log‐transformed *Δ* *C* *q* values. Normality was assessed using the Shapiro–Wilk test, and homogeneity of variances was confirmed using Bartlett′s test. *Δ* *C* *q* values for ACE2 expression were normally distributed (*p* = 0.21), and Bartlett′s test was used to confirm homogeneity of variances (*p* = 0.56).

Accordingly, differences between the groups were analyzed using one‐way analysis of variance (ANOVA) followed by Dunnett′s multiple comparison test to compare the mild and severe groups with the control group.

The 2^−*Δ* *Δ* *C* *q*^ method was applied to calculate relative fold changes with the control group serving as the calibrator.

In contrast, most biochemical and hematological variables did not meet the normality assumption and were therefore analyzed using the Kruskal–Wallis test with Dunn′s multiple comparison test.

Additionally, a generalized linear model (GLM) was constructed in IBM SPSS Statistics (Version 27.0.1) using *Δ* *C* *q* values as the dependent variable and disease group as the main factor. This model was used to assess potential cofounding effects of demographic variables on ACE2 expression. Covariates included sex (categorical variable) and age (continuous variable). Interaction terms (group × age and group × sex) were included to measure effect modification.

Effect sizes were reported using partial eta squared (partial *η* ^2^) for parametric models, epsilon squared (*ε* ^2^) for Kruskal–Wallis tests (calculated from the H‐statistic using the standard formula), Cramér′s *V* for chi‐square analyses, and Cohen′s *f* for one‐way ANOVA (calculated using the Psychometrica effect size calculator: https://www.psychometrica.de/effect_size.html).

The threshold for statistical significance was set at *p* < 0.05. Effect sizes were interpreted according to conventional thresholds: For Cohen′s *f*, values of 0.10, 0.25, and 0.40 represent small, medium, and large effects, respectively; for partial *η* ^2^, values of 0.01, 0.06, and 0.14 indicate small, medium, and large effects; for *ε* ^2^, values of approximately 0.01, 0.08, and ≥ 0.26 indicate small, moderate, and large effects; and for Cramér′s *V*, values of 0.10, 0.30, and ≥ 0.50 correspond to small, medium, and large effects.

Furthermore, this study characterized the biological and molecular parameters of COVID‐19 patients in Iraq using a combined statistical, exploratory, and machine learning–based approach, with a specific focus on gender differences and severity classification. After preprocessing, normalization, and imputation using the missForest algorithm (R, Version 1.5), 10 normalized laboratory parameters were included in the final dataset.

All data handling and modeling were performed using the R programming language (Version 4.2.2). Key software packages utilized included scales (Version 1.3.0) for normalization, caret (Version 7.0‐1) for data splitting and model training, and ggplot2 (Version 3.5.2) for data visualization.

SMOTE (Version 1.4.0) was applied, and oversampling was only used during each cross‐validation fold′s training phase. To avoid data leakage and ensure accurate performance estimation, it was not applied to the entire dataset before splitting. SMOTE was only used for exploratory modeling and not for validated predictive inference due to the extremely small control class (*n* = 2 in the modeling subset).

Extreme gradient boosting (XGBoost) (Version 1.7.11.1) was implemented using the R framework [25]. SHapley Additive exPlanations (SHAP) values (SHAPforxgboost Version 0.1.3 and shapviz Version 0.10.2) were used to assess model interpretability [26].

As no formal a priori power calculation was performed, given the pilot nature and small sample size of the study, all results should be interpreted as exploratory and hypothesis‐generating.

## 4. Results

### 4.1. Baseline Characteristics

**Figure 1:**
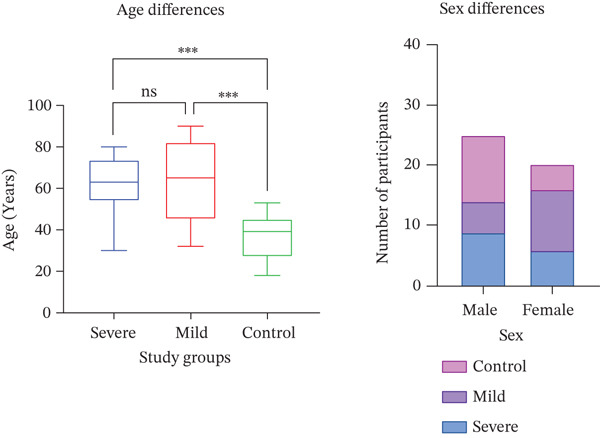
Age and sex distribution in this study. (A) Age distribution across groups. The median and interquartile range are shown in boxplots. There were significant differences in age between the groups (Kruskal–Wallis test, *H* = 18.96, *p* < 0.0001). Both mild and severe patients were significantly older than controls, as determined by post hoc Dunn′s test (adjusted *p* values of 0.0003 and 0.0008, respectively). Still, there was no apparent disparity between the mild and severe groups (ns). (B) Sex distribution across study participants. The number of male and female participants in each severity category is displayed in a bar chart. There was no significant difference in the sex distribution between the groups (*χ* ^2^(2) = 5.040, *p* = 0.0805).  ^∗∗∗^ *p* ≤ 0.001 (very highly significant); *p* > 0.05 (ns, nonsignificant).

Ages varied significantly among the three groups (Figure 1A) (Kruskal–Wallis *H* = 18.96, *p* < 0.0001; *ε* ^2^ = 0.40, large effect). While there was no significant difference between the mild and severe groups (*p* > 0.9999), post hoc Dunn′s test showed that both mild (median = 65 years) and severe (median = 63 years) patients were significantly older than controls (median = 39 years) (adjusted *p* = 0.0008 and *p* = 0.0003, respectively). Table 4 summarizes the baseline demographic characteristics. However, group differences in sex distribution were not statistically significant (Figure 1B) based on chi‐square analysis (*χ* ^2^(2) = 5.040, *p* = 0.0805; Cramér′s *V* = 0.33, small‐to‐moderate effect).

**Table 4:** Baseline characteristics of study participants.

Variable	Severe (*n* = 15)	Mild (*n* = 15)	Control (*n* = 15)	*p*value
Age (years)				
Median (interquartile range or IQR)	63 (54–74)	65 (45–82)	39 (27–45)	< 0.0001 ^∗^
Range	30–80	32–90	18–53	
Sex				
Female, *n* (%)	6 (40.0%)	10 (66.7%)	4 (26.7%)	0.0805 ^∗∗^
Male, *n* (%)	9 (60.0%)	5 (33.3%)	11 (73.3%)	

^∗^ *p* ≤ 0.05 (significant).

^∗∗^ *p* > 0.05 (not significant).

### 4.2. Hematological and Biochemical Alteration Across Clinical Groups

**Figure 2:**
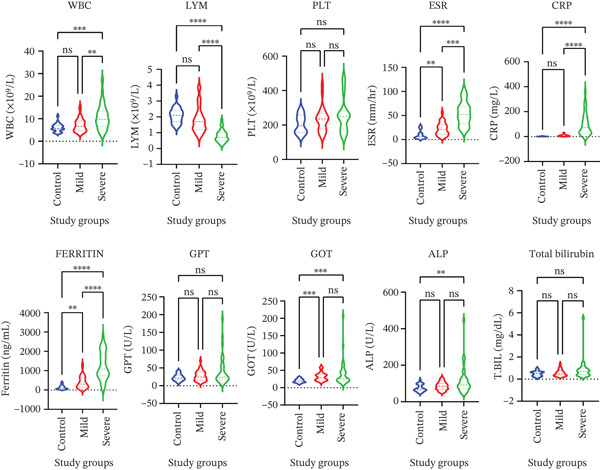
Alterations in hematology and biochemistry in COVID‐19 study groups. Violin plots illustrating the distribution of 10 key hematological and biochemical biomarkers (WBC, LYM, PLT, ESR, CRP, ferritin, GPT, GOT, ALP, and T.BIL) across control, mild, and severe COVID‐19 cohorts.  ^∗∗∗∗^ *p* ≤ 0.0001 (very, very highly significant);  ^∗∗∗^ *p* ≤ 0.001 (very highly significant);  ^∗∗^ *p* ≤ 0.01 (highly significant); *p* > 0.05 (ns, nonsignificant).

A distinct severity‐dependent pattern was observed in the hematological and biochemical profiles (Figure 2), with Table 5 providing statistical significance. Severe disease was characterized by progressive leukocytosis and marked lymphopenia, highlighting immune dysregulation as a key aspect of progression. In advanced cases, inflammatory biomarkers such as ferritin, CRP, and ESR were markedly increased, indicating a more severe systemic inflammatory response. Significant increases in AST and ALP in cases of severe disease also suggested hepatic involvement. Collectively, these findings indicate an increasing inflammatory burden and multisystem involvement as the disease severity progresses. Also, Table 5 shows particularly large effect size estimates for CRP, ferritin, ESR, and lymphocyte counts, supporting the finding of strong group effects.

**Table 5:** Comparison of clinical parameters across study groups including Kruskal–Wallis effect sizes (*ε* ^2^).

Parameter	Control	Mild	Severe	*p*value	Effect size (*ε* ^2^)
Hematological					
WBC (×10^3^/*μ*L)	6.07 ± 1.75	7.19 ± 2.96	10.79 ± 5.49	< 0.001	0.17
LYM (×10^3^/*μ*L)	2.09 ± 0.47	1.80 ± 0.81	0.79 ± 0.37	< 0.0001	0.56
PLT (×10^3^/*μ*L)	204.8 ± 55.44	234.1 ± 73.55	257.3 ± 99.83	ns	0.00
Inflammatory markers					
ESR (mm/h)	9.56 ± 8.54	21.90 ± 13.13	54.37 ± 24.47	< 0.0001	0.44
CRP (mg/L)	4.36 ± 2.76	22.91 ± 35.42	62.81 ± 74.74	< 0.001	0.71
Ferritin (ng/mL)	134.0 ± 107.3	407.1 ± 327.1	1242 ± 717.1	< 0.0001	0.55
Biochemical/liver					
GOT/AST (U/L)	19.94 ± 5.49	31.82 ± 11.71	40.96 ± 37.86	< 0.001	0.18
GPT/ALT (U/L)	23.31 ± 9.31	28.06 ± 14.44	42.49 ± 40.95	ns	0.00
ALP (U/L)	75.04 ± 19.33	85.97 ± 21.96	116.7 ± 77.62	< 0.01	0.08
T.BIL (mg/dL)	0.50 ± 0.23	0.55 ± 0.30	0.86 ± 0.98	ns	0.04

*Note:* Epsilon squared (*ε* ^2^) was used as a measure of effect size for Kruskal–Wallis analyses; values of approximately 0.01, 0.08, and ≥ 0.26 indicate small, moderate, and large effects, respectively.

### 4.3. ACE2 Gene Expression Analysis

**Figure 3:**
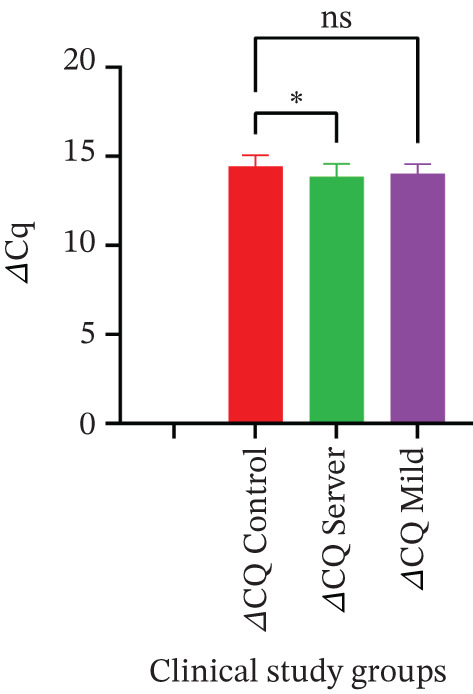
Levels of *Δ* *C* *q* across clinical severity groups. The mean *Δ* *C* *q* values ± standard error of the mean (SEM) for each clinical severity group are displayed in a bar graph. Severe cases exhibited a significant decrease in *Δ* *C* *q* compared to the control ( ^∗^ *p* = 0.0321), indicating that gene expression increases with disease severity. However, no significant difference was observed between mild and healthy controls.

To evaluate variations in gene expression across clinical severity groups, a one‐way ANOVA was performed to compare *Δ* *C* *q* values among the control, mild, and severe groups. The analysis revealed a statistically significant difference between the three groups: *F*(2, 42) = 3.289; *p* = 0.0471, with an *R* ^2^ of 0.1354. The calculated Cohen′s *f* was 0.37, indicating a moderate to large effect size (Figure 3).

Post hoc analysis using Dunnett′s multiple comparison test demonstrated that the severe group had significantly lower *Δ* *C* *q* values compared to the control group (mean difference = 0.5680, 95*%*CI = 0.0437–1.092, adjusted *p* = 0.0321), as detailed in Table 6. No significant difference was observed between the control and mild groups (mean difference in *Δ* *C* *q* = 0.414; 95% CI: −0.110 to 0.938; adjusted *p* = 0.138); however, this should not be interpreted as evidence of equivalence. Importantly, the confidence interval for the severe‐versus‐control comparison excluded zero, supporting a measurable group effect despite the pilot‐scale sample.

**Table 6:** Comparison of ACE2 expression (*Δ* *C* *q* values) between clinical groups.

Comparison	Mean *Δ* *C* *q* (control group)	95% CI of difference	Adjusted *p* value	Significance
*Δ* *C* *q* control vs. *Δ* *C* *q* severe	+0.5680	0.0437 to 1.092	0.0321	Significant ^∗^
*Δ* *C* *q* control vs. *Δ* *C* *q* mild	+0.4140	−0.1103 to 0.9383	0.1376	Not significant

^∗^ *p* ≤ 0.05; *p* > 0.05 (not significant).

It is clearly stated that the expression levels found in Table 6 in severe cases are slightly increased in comparison to the control group. Descriptive statistics by group revealed mean ± SD *Δ* *C* *q* values of 14.43 ± 0.62 in controls, 14.02 ± 0.53 in mild cases, and 13.87 ± 0.71 in severe cases, with decreasing *Δ* *C* *q* values suggesting higher ACE2 expression with increasing disease severity (Table 7).

**Table 7:** ACE2 mRNA expression status across clinical groups. Interpretation derived from statistical results presented in Table 6.

Group	ACE2 expression (relative to control)	Statistical significance
Severe	Higher expression	Significant (*p* = 0.0321)
Mild	Similar expression	Not significant
Control	Baseline	—

The 2^−*Δ* *Δ* *C* *q*^ method was used to calculate relative ACE2 expression and corresponding fold change, with the control group (mean *Δ* *C* *q* = 14.43) as the calibrator. Compared with controls, the mild and severe groups showed fold changes of 1.33 and 1.47, respectively (Table 8), supporting a trend toward elevated relative ACE2 expression with increasing disease severity.

**Table 8:** Relative gene expression analysis (2^−*Δ* *Δ*Ct^ method).

Group	Mean *Δ* *C* *q*	*Δ* *Δ* *C* *q*	Fold change (2^−*Δ* *Δ* *C* *q*^)
Control	14.43	0	1.00
Mild	14.02	−0.41	1.33
Severe	13.87	−0.56	1.47 approximately (1.5‐fold)

### 4.4. Influence of Age and Sex on ACE2 Expression

The impact of age and sex on ACE2 expression (*Δ* *C* *q* values) in the three groups (control, mild, and severe) was assessed using a GLM. Age and sex were included as covariates in the model to adjust for potential cofounding effects. The overall model was significant without interaction terms (*F* = 32.860, *p* < 0.001, *R* ^2^ = 0.767). ACE2 expression was significantly correlated with the disease group (*F* = 27.218, *p* < 0.001, partial *η* ^2^ = 0.576), suggesting a large effect size.

Compared with the severe group (reference category), the control group showed significantly higher *Δ* *C* *q* values (regression coefficient [*B*] = 2.213, *p* < 0.001), indicating lower ACE2 expression. In contrast, no significant difference was observed between the mild and severe groups (*p* = 0.417).

Age (*F* = 2.656, *p* = 0.111, partial *η* ^2^ = 0.062) and sex (*F* = 0.297, *p* = 0.589, partial *η* ^2^ = 0.007) were not independently significant predictors.

To determine whether the association between ACE2 expression and disease severity differed by age or sex, interaction terms were included. The overall interaction model was significant, and a significant group × age interaction was observed (*F* = 27.567, *p* < 0.001, partial *η* ^2^ = 0.691), representing a large effect.

Specifically, in the severe group, increasing age was associated with lower *Δ* *C* *q* values (*B* = −0.034, *p* = 0.001), indicating higher ACE2 expression with advancing age. In contrast, no significant group × sex interaction was detected (*F* = 1.179, *p* = 0.331, partial *η* ^2^ = 0.087), and the sex × age interaction was also not significant (*F* = 1.745, *p* = 0.195, partial *η* ^2^ = 0.045).

### 4.5. Bioinformatic Findings From Gender and Severity Classification Analyses

#### 4.5.1. Exploratory Data Analysis (EDA)

**Figure 4:**
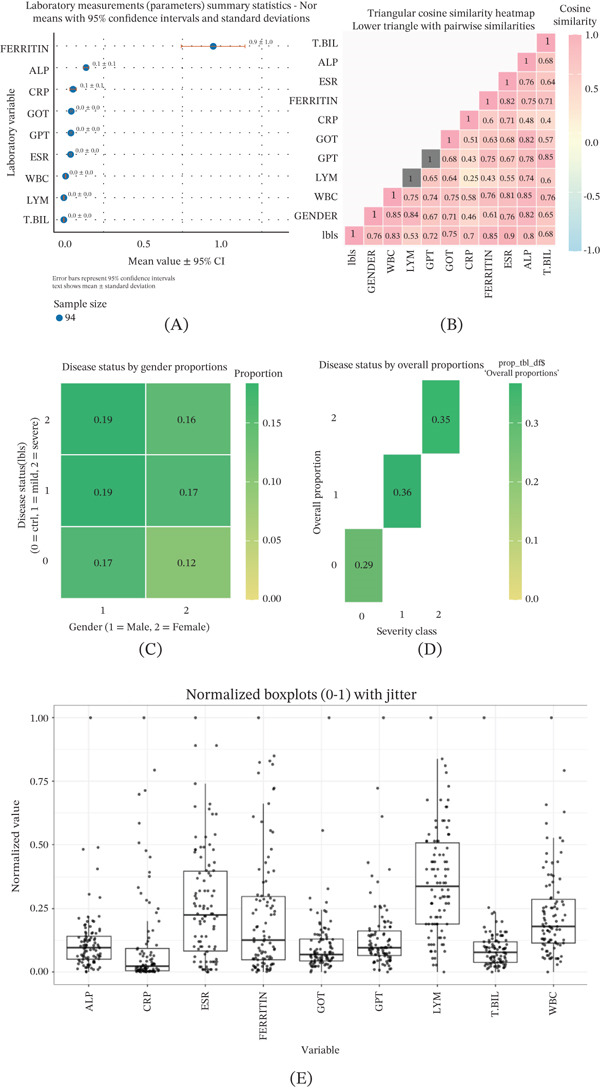
(A–E) Exploratory data analysis (EDA) for laboratory parameters. (A) For all laboratory variables (*n* = 94), summary statistics (mean ± 95*%*CI) showed that ferritin was the most elevated parameter with the widest dispersion, while total bilirubin (T.BIL) was the lowest with minimal variance. (B) The cosine similarity heatmap showed only positive correlations; the strongest were between ESR and severity (cos = 0.90) and between ESR and ferritin (cos = 0.82). (C, D) Cross‐tabulations verified uniform representation across disease status and a proportionate gender distribution across severity categories. (E) Jitter‐added normalized boxplots showed clear patterns of variability, especially elevated dispersion in ferritin and ESR.

EDA identified distinct patterns across the studied parameters (Figure 4A–E). Ferritin exhibited the highest mean value with a sparse distribution, whereas total bilirubin (T.BIL) showed the lowest mean with a dense distribution. Cosine similarity analysis (Figure 4B) revealed an absence of negative correlations; instead, strong positive correlations were observed between several parameters, most notably ESR–ferritin (cos = 0.82) and ESR–severity (cos = 0.90). Cross‐tabulations (Figure 4C,D) confirmed a balanced gender distribution across severity groups, with no missing values remaining after *missForest* imputation.

#### 4.5.2. Multiclass Classification of COVID‐19 Severity

*Δ* *C* *q* values from the qPCR analysis were integrated into the dataset for the multiclass classification of COVID‐19 severity. To address the significant class imbalance (control = 2, mild = 9, and severe = 13), the synthetic minority oversampling technique (smotefamily, Version 1.4.0) was applied. This resulted in a synthetically balanced dataset (*n* = 44) for exploratory modeling.

**Figure 5:**
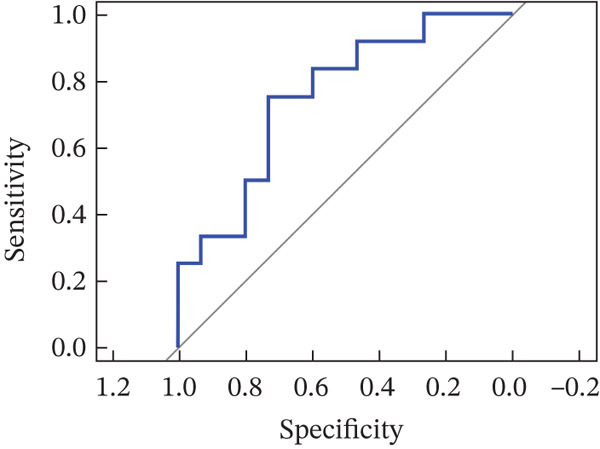
Fitting an extreme gradient boost (XGB) classifier. The XGBoost model performed moderately well (AUC–ROC = 0.76). The sensitivity‐versus‐specificity trade‐off is visualized by the ROC curve.

**Figure 6:**
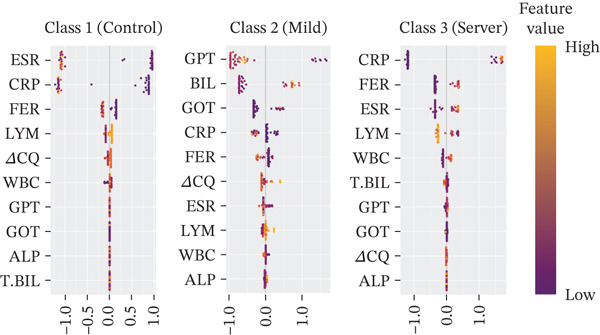
Class‐specific SHAP analysis for COVID‐19 severity. In the multiclass classification (control, mild, and severe), SHAP summary plots showed that ESR, CRP, and ferritin showed higher relative contributions for the control group. In contrast, GPT, T.BIL, and GOT showed higher relative contributions in mild cases, whereas CRP, ferritin, and ESR showed greater relative significance in severe cases.

In this preliminary, hypothesis‐generating analysis, the XGBoost model achieved an apparent classification accuracy of 75%, as illustrated in Figure 5. However, these findings should be interpreted with caution and are not regarded as validated predictive performance due to the small initial control sample size; they require further validation in larger, independent cohorts. According to SHAP analysis (*shapviz* package), the most influential factors varied by group:

• Control group: ESR, CRP, and ferritin were the primary contributors.
• Mild group: GPT, T.BIL, and GOT were the most significant factors.
• Severe group: CRP, ferritin, and ESR demonstrated the greatest relative contributions (Figure 6).

**Figure 7:**
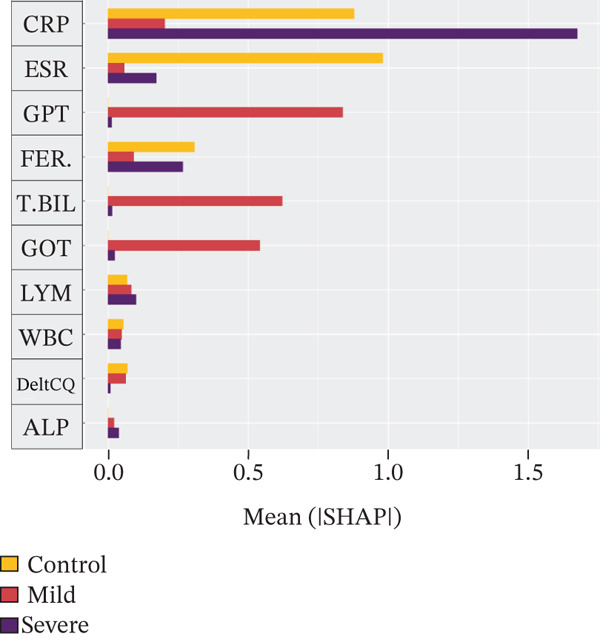
Global feature importance across severity classes. In the exploratory model, mean absolute SHAP values indicated that CRP, ESR, and GPT had relatively higher contributions across severity categories, with CRP showing greater relative importance in severe classification.

In global feature importance plots (Figure 7) from the exploratory model across severity classes, CRP, ESR, and GPT showed the greatest relative SHAP contributions.

The use of a gradient boosting model carries an inherent risk of overfitting due to the relatively small dataset (*N* = 94; external test set, *N* = 27). Despite the application of repeated cross‐validation and regularization, the observed variability across folds (ROC range 0.25–1.00) indicates instability; therefore, performance should not be interpreted as deployable or clinically validated.

## 5. Discussion

In the present study, several hematological and biochemical indicators shifted significantly in correlation with disease severity. WBC counts, CRP, ESR, ferritin, AST/GOT, and ALP levels were significantly elevated, while lymphocyte levels were significantly lower in patients with severe COVID‐19. Although upward trends were observed for PLT, ALT/GPT, and T.BIL, these results did not reach statistical significance.

The presence of elevated WBC and CRP levels is characteristic of an acute phase response, consistent with our observation. This is supported by Ferdousi et al., who reported that CRP and ferritin levels were significantly elevated in severe and critical COVID‐19 cases [27]. Additionally, we demonstrated that WBC counts rose as severity increased, while lymphocyte counts decreased—a hallmark of SARS‐CoV‐2‐induced immune dysregulation. This lymphopenia is predominantly driven by lymphocyte depletion and programmed cell death triggered by the viral infection.

The severe group in our study showed significantly high ferritin levels, consistent with the hyperferritinemia typically associated with cytokine storm syndrome. The combination of elevated ferritin, ESR, and CRP reinforces the role of ferritin as a robust prognostic biomarker for poor clinical outcomes. This aligns with the findings of Ferdousi et al., who also noted increasing ferritin levels associated with disease severity [27]. Furthermore, ESR steadily rose from the control group to mild cases and peaked in severe patients, directly reflecting disease progression within our sample. According to Feng et al., ESR and CRP were much more variable in COVID‐19 patients with liver injury than in those without, indicating a close connection between inflammation and hepatic dysfunction [28].

In our study, AST and ALP levels differed significantly across severity groups, with ALP levels increasing substantially in severe cases. This corresponds to the findings of Kumar et al., who reported an ALP value of 434 U/L as a marker of liver involvement [29]. In the study by Nobin et al., ALT and AST were both significantly associated with disease severity; however, only a nonsignificant upward trend in ALT was observed across severity groups in our study. In line with our findings, ALP and AST were more commonly elevated than ALT in moderate‐to‐severe cases [29]. Moreover, while there was no statistically significant difference in T.BIL within our study population, an upward trend was observed in severe cases. This is consistent with reports of mild hyperbilirubinemia in patients with COVID‐19‐related liver injury, where levels typically increase while remaining within or near normal reference ranges. Collectively, inflammatory biomarkers (CRP, ESR, and ferritin), lymphopenia, and liver enzyme alterations represent key indicators of severity‐associated signals in this cohort [28].

Our study demonstrated significantly higher ACE2 mRNA expression in severe COVID‐19 patients than in healthy controls (*Δ* *C* *q* = 0.568, *p* = 0.0321), indicating transcriptional upregulation with disease severity. No significant difference was observed between mild cases and controls; however, this should not be interpreted as evidence of equivalence. The severe‐versus‐control contrast was observed with a moderate effect size (Cohen′s *f* = 0.37) and a confidence interval excluding zero, indicating that the observed signal is unlikely to be solely attributable to sampling variability; however, it should be interpreted with caution given the pilot sample size.

These outcomes are consistent with observations of elevated ACE2 transcription in severe COVID‐19 patients. For instance, a study by Han et al. demonstrated that ACE2 is upregulated in respiratory tissues during SARS‐CoV‐2 infection, driven by SP1‐induced transcription and the PI3K/AKT pathway in human lung epithelial cells [30]. Moreover, Wang et al. showed that SARS‐CoV‐2 induces increases in both mRNA and protein expression of ACE2 to facilitate viral entry, further supporting the concept of transcriptional enhancement during infection [31].

Some studies, however, have reported decreased ACE2 expression in peripheral immune cells during COVID‐19, which contrasts with our findings. ACE2 was found to be downregulated in PBMCs from infected patients [32, 33]. In a more recent study, Barreto Fernandes et al. found that PBMCs from hospitalized patients who needed oxygen supplementation exhibited lower ACE2 expression [34]. The biological matrix examined may account for some of the discrepancies between our results and earlier reports. Our analysis was performed on whole blood RNA, which includes additional cellular compartments and may reflect broader systemic transcriptional responses, whereas these studies assessed isolated PBMCs. More accurate information on compartment‐specific ACE2 regulation could be obtained by isolating specific leukocyte subsets or by using single‐cell transcriptomic approaches; however, these analyses were beyond the scope of the current investigation.

The modest effect size of the ANOVA model (*R* ^2^ = 0.135) indicates that only a small proportion of the variation in ACE2 expression is explained by disease severity. This result implies that biological processes other than clinical severity also affect ACE2 regulation. Within the renin–angiotensin–aldosterone system (RAAS), ACE2 produces Ang (1–7), which communicates via the Mas1 receptor (MAS1) to counteract the proinflammatory ACE/Ang II axis [35]. Therefore, changes in ACE2 expression should be interpreted within the broader context of coordinated regulation of MAS1, ACE, and other RAAS components. Furthermore, TMPRSS2 coexpression is required for viral entry, indicating that disease severity may not be determined solely by ACE2 levels [36].

Moreover, ACE2 expression was measured using the whole blood RNA from the buffy coat fraction, a heterogeneous population of circulating leukocytes that includes monocytes and lymphocytes. Variations in leukocyte composition may partially influence *Δ* *C* *q* values because lymphopenia and changes in leukocyte composition characterize severe COVID‐19. Although leukocyte differences were noted, the ACE2 expression of isolated cellular subsets was not assessed. Therefore, the elevated ACE2 expression observed in severe cases likely reflects a combination of transcriptional regulation and shifts in immune cell composition.

Blood samples were collected at the time of the clinical presentation. Severe cases were sampled upon ICU admission due to critical clinical status, whereas mild cases were assessed during routine hospital evaluation. Consequently, ACE2 expression most likely reflects variations in disease severity at presentation rather than longitudinal progression. This elevation in severe cases might be associated with the RAAS dysregulation and systemic inflammation, which are hallmarks of critical illness.

To address potential confounding factors and differences in age distribution, we first assessed the baseline characteristics of our cohort. Using the Kruskal–Wallis test and one‐way ANOVA, we found that both mild and severe patients were significantly older than healthy controls (*p* < 0.05). Since age‐related immune and inflammatory changes can influence ACE2 levels, this age imbalance was carefully considered during our interpretation. Notably, there was no discernible age difference between the mild and severe groups, indicating that age alone is unlikely to be the primary factor driving the variation in ACE2 expression observed across severity categories.

To rigorously validate this, a GLM was applied to adjust ACE2 expression (*Δ* *C* *q* values) for age and sex. Our findings indicate that, although age and sex were not independent predictors of ACE2 expression (*p* = 0.111 and *p* = 0.589, respectively), “group” remained a significant predictor with a large effect size (partial *η* ^2^ = 0.576, *p* < 0.001). Furthermore, effect sizes for sex‐related terms were minimal, and sex did not significantly alter the relationship between ACE2 expression and disease severity (group × sex interaction, *p* = 0.331).

Most importantly, our interaction analysis revealed a significant group × age interaction (*p* < 0.001), representing a large effect size. Specifically, in the severe COVID‐19 group, advancing age was associated with even higher ACE2 expression (lower *Δ* *C* *q* values, *B* = −0.034, *p* = 0.001). Importantly, this suggests that age may amplify ACE2‐related responses in advanced stages of the disease, potentially explaining why older patients are more susceptible to severe outcomes. Overall, these results demonstrate that ACE2 expression is severity‐dependent and synergistically influenced by age, but is not significantly impacted by sex.

Previous studies have reported mixed findings regarding ACE2 expression and demographic characteristics. In human lung tissues, age‐related variation in ACE2 expression has been observed: Older individuals have more ACE2‐expressing alveolar Type II cells, suggesting that age may affect susceptibility to severe SARS‐CoV‐2 infection [22]. Also, in another study, ACE2 expression in the nasal epithelium has been shown to increase with age, with children exhibiting noticeably lower expression than adults [37]. However, other papers found that ACE2 expression is not strongly correlated with demographic variables such as age, but rather varies primarily among respiratory cell types. A thorough examination of human respiratory tissues revealed that ACE2 expression varied among airway epithelial cells and lacked a clear age‐dependent pattern [38]. Given that ACE2 is found on the X chromosome and may be influenced by sex hormones, males and females may express ACE2 differently [21]. In fact, some clinical studies have found that men have higher levels of circulating ACE2 [39]. On the other hand, after controlling for covariates, our results revealed no significant independent effect of sex on ACE2 expression. This is consistent with multiple transcriptomic analyses that found no significant sex‐related variation in ACE2 expression across tissues [40].

According to multivariate and machine learning analyses, ACE2 expression is not a dominant predictor of severity, despite being slightly but significantly elevated in severe cases. Conversely, stronger predictive contributions were observed for inflammatory biomarkers such as ferritin, CRP, and ESR. These results imply that rather than serving as a stand‐alone predictor of clinical severity, ACE2 may serve as an additional molecular marker. Before clinical translation, our whole blood results must be validated in larger, tissue‐specific studies, given contradictory evidence regarding ACE2 regulation: It has been reported to be upregulated in respiratory tissues but downregulated in circulating PBMCs [31–34].

In this study, XGBoost, a regularized tree‐based ensemble technique intended to enhance predictive performance and manage overfitting, was used for exploratory modeling [25], in which ferritin, CRP, ESR, GPT, GOT, and T.BIL were found to have higher relative SHAP contributions in exploratory modeling. Notably, ferritin, CRP, and ESR were most predictive of severe disease, while GPT, T.BIL, and GOT were more prevalent in mild cases. SHAP, which measures feature contributions to model predictions, was used to evaluate the model′s interpretability [26]. SHAP interaction plots showed that ferritin and ESR, and ferritin and GPT, had synergistic effects. Significant correlations (*r* = 0.82) were found between ESR and ferritin, suggesting that inflammatory markers are multicollinear. Instead of distinct biological pathways, high multicollinearity suggests that these variables reflect a common inflammatory signal. Because correlated predictors may compete in the model, resulting in unstable importance hierarchies, individual feature rankings from SHAP analysis should be interpreted with caution. These results support the idea that severity classification in this cohort is based on systemic inflammation rather than a single biomarker. The synergistic relationship between ferritin and ESR may reflect shared inflammatory pathways and suggests that combined measurement may improve prognostic value compared to individual markers. Individual feature rankings should be interpreted with caution because high multicollinearity among inflammatory markers indicates a shared biological signal.

These findings concur with several studies identifying elevated ferritin, CRP, and ESR as key indicators of hyperinflammation and a poor prognosis in COVID‐19 [41, 42]. Additionally, evidence suggests that abnormalities in liver enzymes are closely associated with systemic inflammation and overall disease severity [43–45].

However, other research indicates that CRP may be less specific for COVID‐19 severity when concurrent inflammatory conditions are present [46]. Similar results were reported by Ye and Song [47], who found that liver enzyme abnormalities were generally mild and did not differ significantly between mild and severe COVID‐19 cases. Accordingly, more extensive, controlled studies are required to confirm these biomarker associations. Because of the small dataset and observed cross‐validation variability, these modeling results should be interpreted as hypothesis‐generating rather than clinically validated.

Several limitations should be acknowledged. First, the comparison of ACE2 expression levels between mild and severe patients was based on a small sample size; therefore, these results must be validated in larger cohorts. Given the pilot sample size of 45, the multivariate and machine learning analyses should be considered exploratory and hypothesis‐generating rather than confirmatory. Validation in larger, independent groups will be necessary to establish robust and generalizable predictive performance. Second, the cross‐sectional design provides only a single‐time assessment; consequently, it is not possible to evaluate dynamic changes in biomarkers and gene expression over the course of the disease. Furthermore, direct comparisons with existing literature are complicated by the possibility that whole blood ACE2 measurements may not accurately reflect tissue‐specific expression patterns documented in other studies. Measuring ACE2 in whole blood may not reflect its expression in the respiratory epithelium, where the virus enters, thereby limiting its biological relevance. To confirm and build on these results, longer term, isoform‐specific research with larger and more diverse cohorts is required. Also, care must be taken when interpreting the use of SMOTE for severity classification, given the small sample size in the control group. In such a small minority class, synthetic oversampling could limit generalizability and introduce instability. These modeling results are therefore considered exploratory and need to be confirmed in larger cohorts. Lastly, the primer set employed in this study detects total ACE2 transcripts but does not differentiate between the canonical full‐length ACE2 and the interferon‐inducible truncated isoform (dACE2). Isoform‐specific assays will be necessary in future studies to clarify the distinct contributions of each isoform.

## 6. Conclusion

The current study demonstrates that systemic inflammatory responses, as indicated by elevated CRP, ESR, ferritin, and altered hematological parameters, are the main drivers of COVID‐19 severity. Compared with inflammatory biomarkers, the ACE2 gene′s contribution to disease classification was minimal, despite its expression varying across severity groups. These results suggest that rather than ACE2 expression alone, COVID‐19 severity is impacted by complex interactions between inflammatory pathways and components of the RAAS. Moreover, age varied between patients and controls. Still, there was no discernible age difference between mild and severe cases, indicating that the observed variations in disease severity were unlikely to be explained by demographic factors alone. Nevertheless, larger multicenter studies are warranted to validate these findings and to explore the clinical significance of ACE2 as a prognostic tool or therapeutic target in the management of COVID‐19. These findings suggest potential associations between inflammatory biomarkers and disease severity that warrant further investigation.

## Author Contributions

P.J.J. conceived and designed the study. K.R.A.S. performed clinical data collection and laboratory measurements, conducted the statistical analysis, interpreted the data, and drafted the manuscript. P.J.J. supervised the project and provided critical revisions to the manuscript.

## Funding

No funding was received for this manuscript.

## Disclosure

Both authors have approved the final version and agree to be accountable for all aspects of the work.

## Conflicts of Interest

The authors declare no conflicts of interest.

## Supporting information


**Supporting Information** Additional supporting information can be found online in the Supporting Information section. File S1: Melting curve analysis and instrument output reports of representative RT‐qPCR runs. File S2: Raw *Δ* *C* *q* calculations and ACE2 expression values for control, mild, and severe groups. File S3: Demographic and clinical data of participants for exploratory analyses. File S4: Additional machine learning outputs, including gender classification, SHAP interaction analyses, and force plots. File S5: List of reagents, instruments, manufacturers, and catalog numbers used in laboratory procedures.

## Data Availability

The data supporting the findings of this study are available from the corresponding author upon reasonable request. These data are not publicly available due to privacy or ethical restrictions.
