# Supplementary material for: ACE2 Expression and Clinical Biomarkers in COVID‐19: Associations With Disease Severity
Source: Pulm Med. 2026 Jun 10;2026:1013605. doi: 10.1155/pm/1013605 (PMC13254222; doi:10.1155/pm/1013605)
Supplement: Supplementary file 1 — Supporting Information Additional supporting information can be found online in the Supporting Information section. File S1: Melting curve analysis and instrument output reports of representative RT‐qPCR runs. File S2: Raw Δ C q calculations and ACE2 expression values for control, mild, and severe groups. File S3: Demographic and clinical data of participants for exploratory analyses. File S4: Additional machine learning outputs, including gender classification, SHAP interaction analyses, and force plots. File S5: List of reagents, instruments, manufacturers, and catalog numbers used in laboratory procedures. [file PM-2026-1013605-s001.docx]

**Supplementary file**

1. **Melting curve analysis and instrument output reports of representative RT-qPCR runs.**


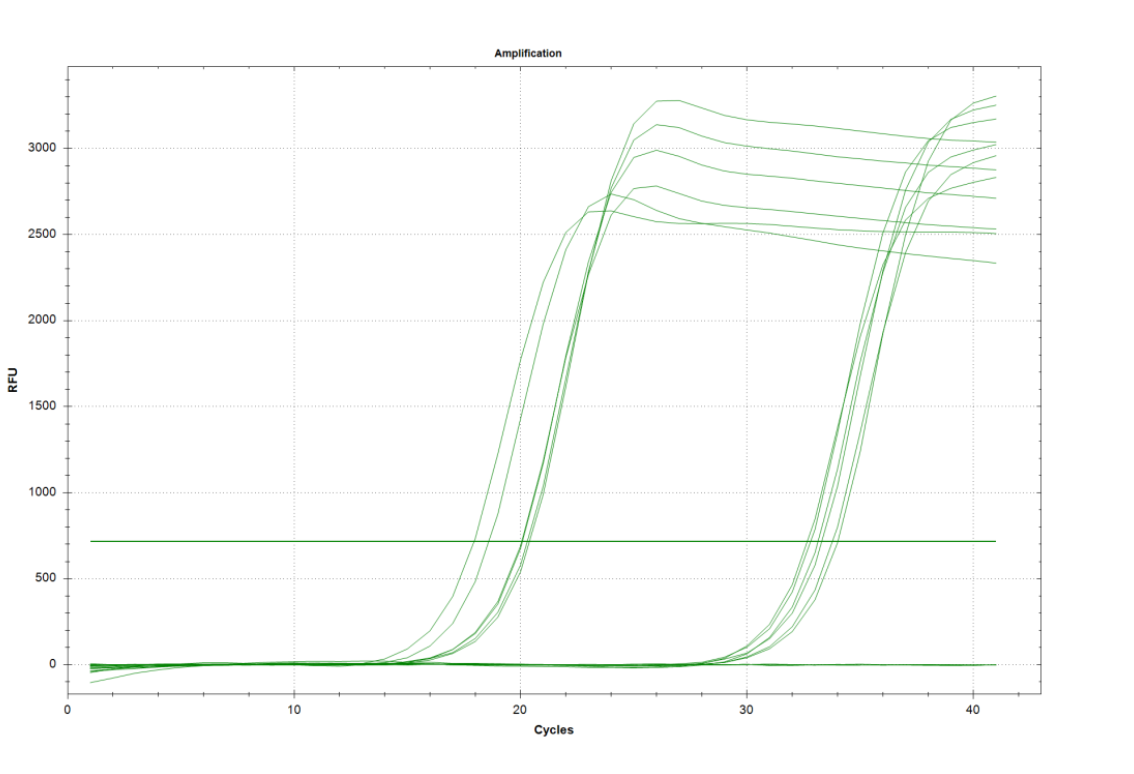


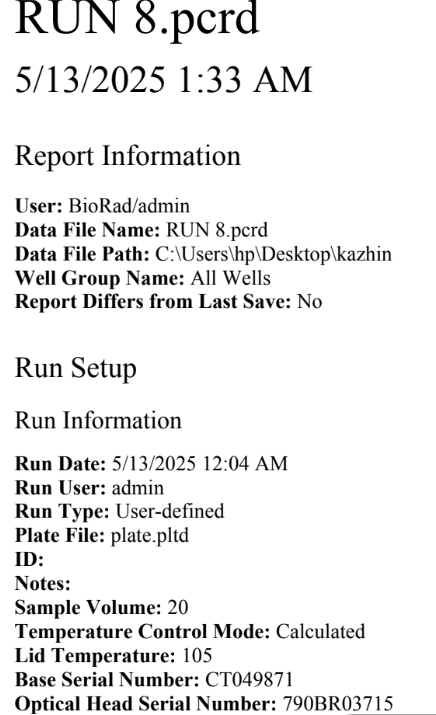


| 1. **Raw ΔCq calculations and ACE2 expression values for control, mild, and severe groups.**  \| Code/MILD \| SAMPLE/mild \| ∆ CQ MILD \| GAPDH/mild \| \| --- \| --- \| --- \| --- \| \| ACE2 1 \| 32.26 \| 14.32 \| 17.94 \| \| ACE2 2 \| 31.52 \| 14.3 \| 17.22 \| \| ACE2 3 \| 31.57 \| 13.59 \| 17.98 \| \| ACE2 5 \| 31.44 \| 13.69 \| 17.75 \| \| ACE2 6 \| 31.19 \| 13.39 \| 17.8 \| \| ACE2 7 \| 30.84 \| 13.26 \| 17.58 \| \| ACE2 8 \| 31.52 \| 14.61 \| 16.91 \| \| ACE2 9 \| 32.02 \| 14.48 \| 17.54 \| \| ACE2 10 \| 31.57 \| 14.67 \| 16.9 \| \| ACE2 12 \| 31.11 \| 13.57 \| 17.54 \| \| ACE2 13 \| 31.01 \| 13.3 \| 17.71 \| \| ACE2 14 \| 31.67 \| 13.78 \| 17.89 \| \| ACE2 16 \| 32.5 \| 14.57 \| 17.93 \| \| ACE2 17 \| 31.66 \| 14.08 \| 17.58 \| \| ACE2 18 \| 32.01 \| 14.69 \| 17.32 \| | | | | |
| --- | --- | --- | --- | --- | --- | --- | --- | --- | --- | --- | --- | --- | --- | --- | --- | --- | --- | --- | --- | --- | --- | --- | --- | --- | --- | --- | --- | --- | --- | --- | --- | --- | --- | --- | --- | --- | --- | --- | --- | --- | --- | --- | --- | --- | --- | --- | --- | --- | --- | --- | --- | --- | --- | --- | --- | --- | --- | --- | --- | --- | --- | --- | --- | --- | --- | --- | --- | --- |
| code/CONTROL | sample/control | GAPDH/control | ∆ CQ CONTROL |  |
| ACE2 19 | 31.38 | 18.22 | 13.16 |  |
| ACE2 20 | 31.87 | 17.64 | 14.23 |  |
| ACE2 37 | 30.07 | 15.95 | 14.12 |  |
| ACE2 38 | 30.39 | 15.06 | 15.33 |  |
| ACE2 39 | 30.68 | 16.83 | 13.85 |  |
| ACE2 40 | 30.24 | 15.75 | 14.49 |  |
| ACE2 41 | 31.03 | 17.27 | 13.76 |  |
| ACE2 42 | 30.42 | 16.08 | 14.34 |  |
| ACE2 43 | 30.35 | 16.41 | 13.94 |  |
| ACE2 44 | 31.46 | 16.13 | 15.33 |  |
| ACE2 45 | 30.53 | 16.15 | 14.38 |  |
| ACE2 46 | 31.06 | 16.06 | 15 |  |
| ACE2 47 | 30.82 | 16.09 | 14.73 |  |
| ACE2 48 | 31.11 | 16.45 | 14.66 |  |
| ACE2 49 | 30.95 | 15.76 | 15.19 |  |

| CODE/SEVERE | SAMPLE/SEVERE | ∆ CQ SEVERE | GAPDH/SEVERE |
| --- | --- | --- | --- |
| ACE2 22 | 31.11 | 15.08 | 16.03 |
| ACE2 23 | 30.98 | 13.18 | 17.8 |
| ACE2 24 | 30.95 | 13.56 | 17.39 |
| ACE2 25 | 31.06 | 13.84 | 17.22 |
| ACE2 26 | 30.71 | 14.42 | 16.29 |
| ACE2 27 | 32.56 | 13.45 | 19.11 |
| ACE2 28 | 31.71 | 14.43 | 17.28 |
| ACE2 29 | 31.18 | 14.94 | 16.24 |
| ACE2 30 | 31.1 | 13.84 | 17.26 |
| ACE2 31 | 31.07 | 13.64 | 17.43 |
| ACE2 32 | 30.43 | 13.85 | 16.58 |
| ACE2 33 | 30.56 | 14.08 | 16.48 |
| ACE2 34 | 31.75 | 12.53 | 19.22 |
| ACE2 36 | 30.68 | 14.31 | 16.37 |
| ACE2 50 | 32.31 | 12.84 | 19.47 |

1. **Participant demographic and clinical data used in exploratory analyses.**


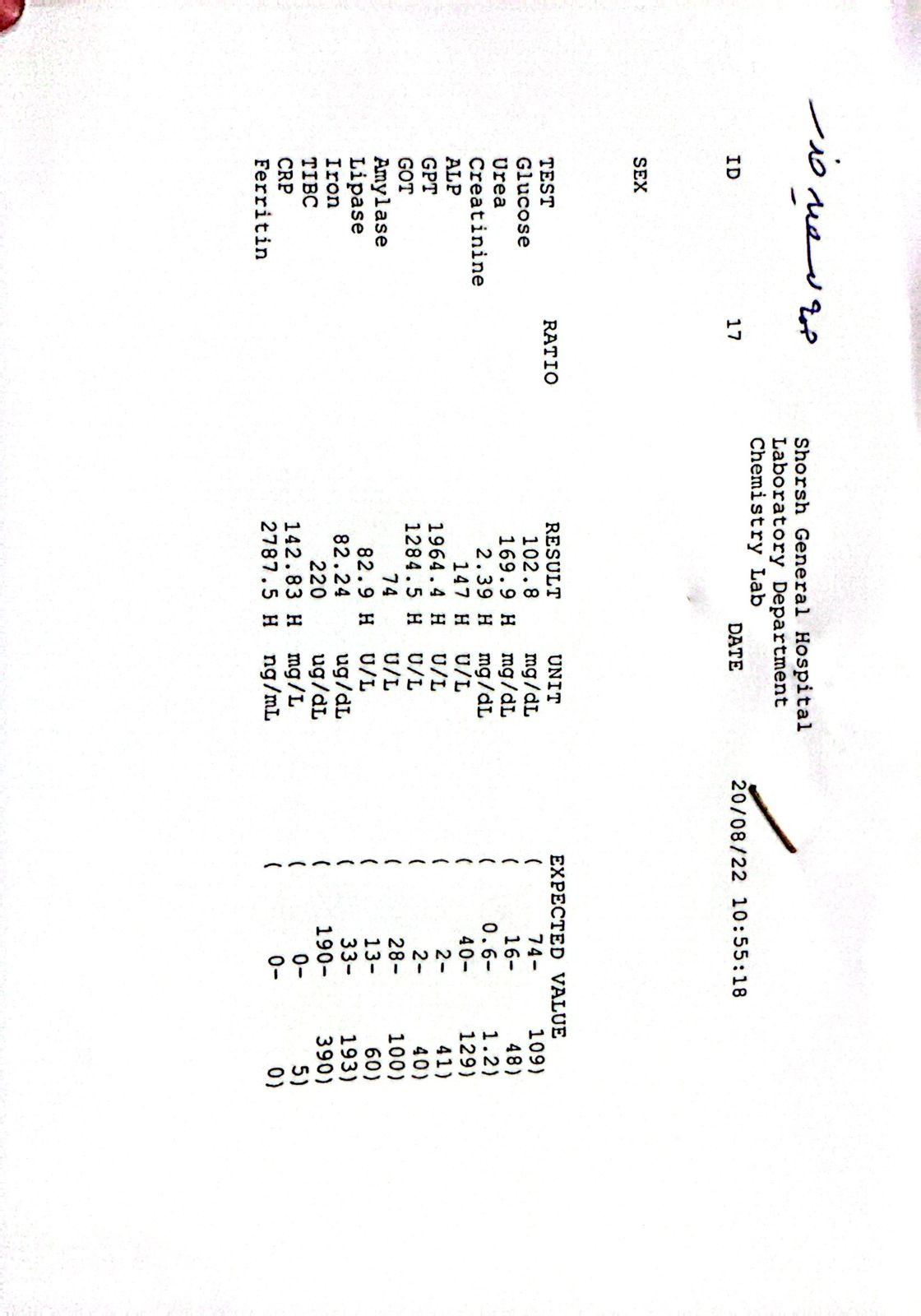


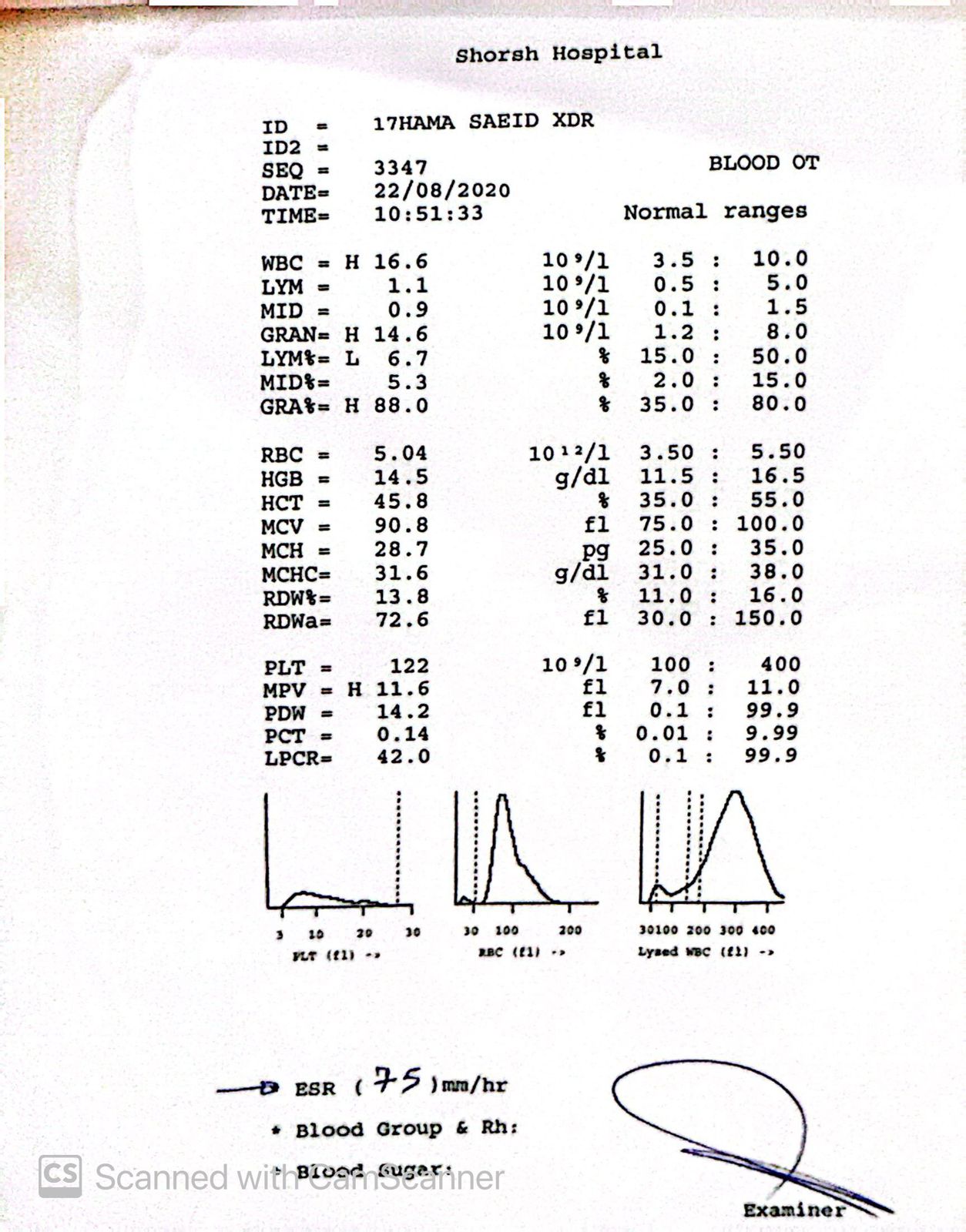


1. **Additional machine learning outputs, including gender classification, SHAP interaction analyses, and force plots.**

**
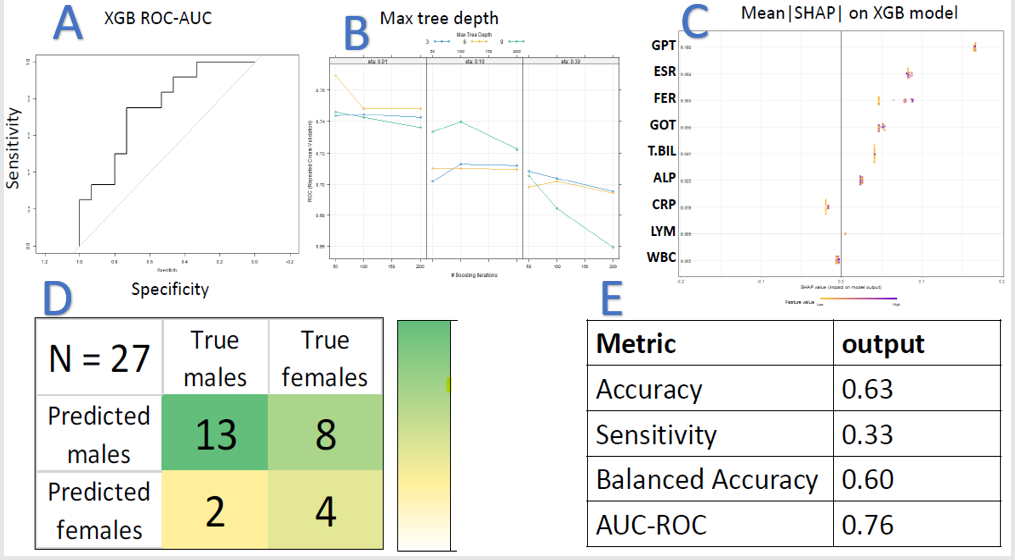
**

B,D, E removed from main manuscript

**4.3.2 Binary classification based on gender**

First attempts at gender-based binary classification were made using regularised GLM-net logistic regression and logistic regression (Figures 4A–D), with balanced accuracies of 0.46–0.48 and accuracies of 0.52 and 0.54, respectively. These results indicated sub-optimal discrimination between males and females. With an accuracy of 0.63 and an AUC-ROC of 0.76, the *xgboost* model (Figure 5E) considerably enhanced classification performance. According to SHAP analysis, GPT, ESR, and FER are the most critical parameters affecting the model's decision-making process, and are used to distinguish between the two classes (Male and Female) (Figure 4C). Further data was revealed by SHAP pairwise dependency (interaction) plots (Figure 6), which demonstrated a strong positive interaction between Ferritin and GPT (cos = 0.75), where increases in one were linked to increases in the other. Further interactions between Ferritin and ESR and between ESR and GPT suggested that some biomarkers work together to influence model decisions rather than acting independently to predict gender. The interconnectedness of the top predictive features was further supported by these interaction trends, which were in line with the cosine similarity matrix results from Figure 3B.


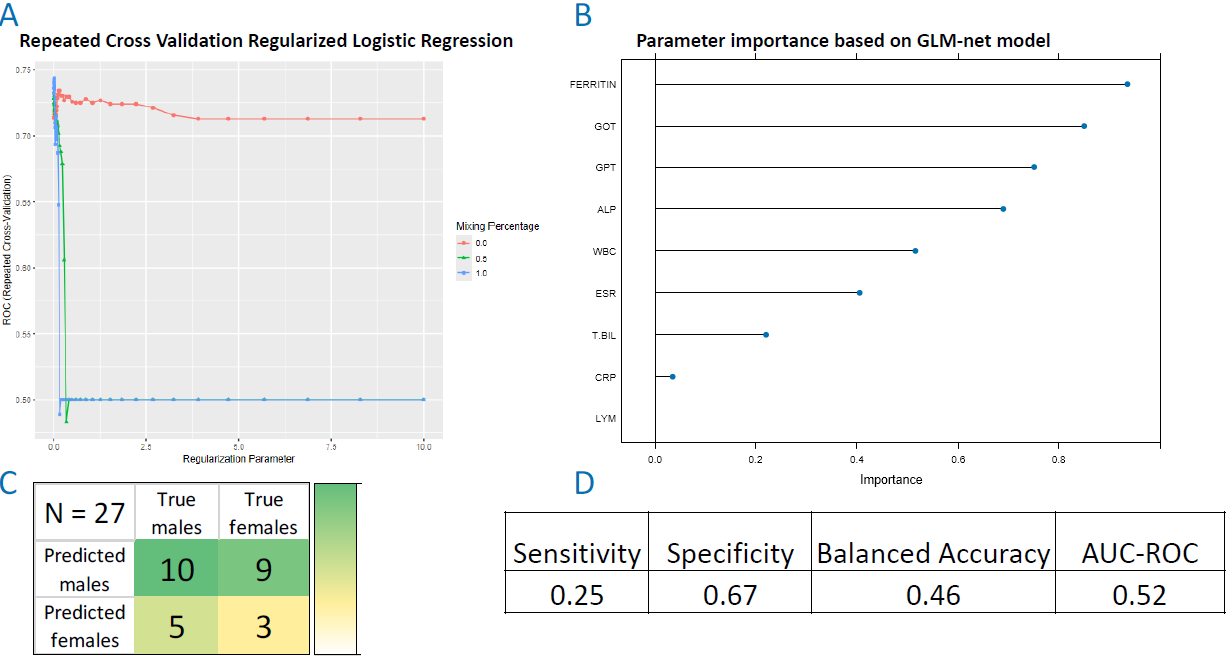


**Figure 4 (A-D):** **Fitting regularised logistic regression model for gender.**

(A) Under repeated cross-validation, ROC performance trends showed marginal predictive stability across regularisation strengths. (B) Ferritin, GOT, and GPT were found to be the main contributors to the GLM-net model by feature importance analysis. (C) The confusion matrix showed balanced accuracy (0.46) and limited sensitivity (0.25). (D) Performance metrics with an AUC–ROC of 0.52 validated suboptimal discrimination.

**4.3.2.1 SHAP-Based Gender Interaction Analysis.**

The SHAP pairwise dependency plots illustrate the contribution of biomarker interactions with gender to the classification model (Figure 6). Ferritin was consistently the most prominent interacting parameter among all features, exhibiting strong nonlinear dependencies with hepatic and inflammatory markers. While ESR and ALP exhibited more intricate U-shaped trajectories, indicating that their effects differed across Ferritin strata, rising Ferritin levels significantly increased the predictive contribution of GPT and GOT. As Ferritin increased, CRP showed a consistent upward trend in predictive value, confirming its function as an inflammatory cofactor. Total bilirubin (T.BIL), on the other hand, showed little interaction with gender and stayed relatively constant across Ferritin levels. For immune cell parameters, additional nonlinear effects were noted; WBC and lymphocyte counts made a minor contribution but displayed noticeable changes at higher values. Additionally, ALP showed an increasing contribution at elevated ranges, highlighting its possible influence on predictive outcomes related to gender.

All of these results suggest that Ferritin plays a key role in modulating gender-specific biomarker interactions, thereby establishing a connection between hepatic dysfunction and systemic inflammation in determining the severity of the disease. Ferritin is supported as an integral feature within the model by the nonlinear dependencies, which further highlight the gender-specific, heterogeneous contributions of inflammatory and hepatic pathways.


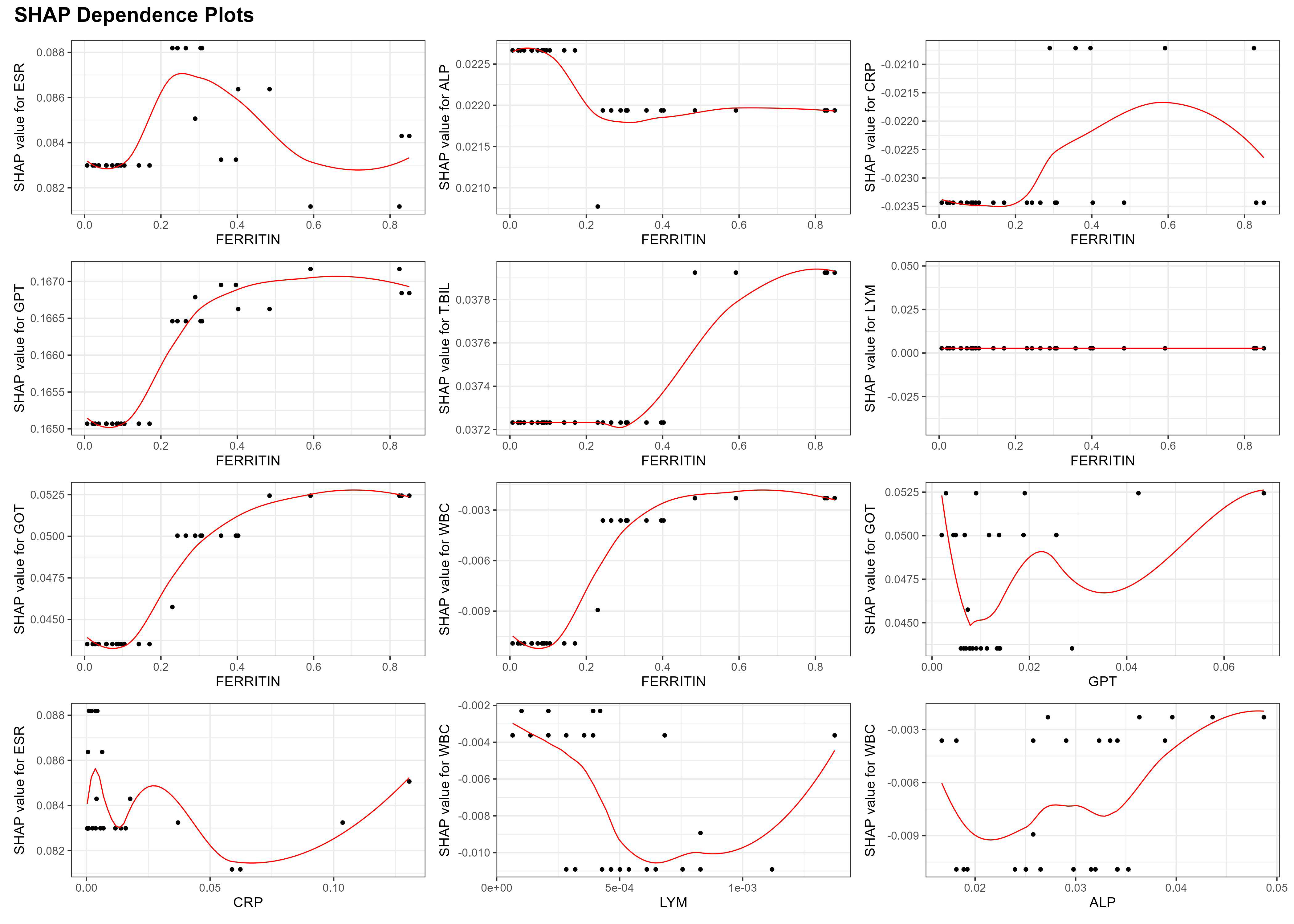


**Figure 6**: **SHAP Dependence interaction plots of parameters between the two genders.**

The interaction effects of gender and clinical parameters on model predictions are depicted in the SHAP pairwise dependency plots. With consistent nonlinear relationships to ESR, ALP, CRP, GPT, T.BIL, GOT, and WBC, Ferritin became a key interacting feature.

These results were supported by descriptive statistics (Table 6), which revealed a low lymphocyte count (1.5 × 10⁹/L) and a significantly elevated mean Ferritin (625.0 ng/mL) in comparison to reference ranges, which were in line with documented COVID-19 pathophysiological patterns.

**Table 6: Measures of central tendency (Descriptive statistics)**

| **Variables** | **Mean** | **Count** | **Sem** |
| --- | --- | --- | --- |
| WBC | 8.2 | 94 | 0.4 |
| LYM | 1.5 | 94 | 0.1 |
| GPT | 31.0 | 94 | 2.8 |
| GOT | 31.5 | 94 | 2.4 |
| CRP | 39.5 | 94 | 7.2 |
| Ferritin | 625.0 | 94 | 68.4 |
| ESR | 28.5 | 94 | 2.3 |
| ALP | 93.8 | 94 | 5.3 |
| T.BIL | 0.6 | 94 | 0.1 |


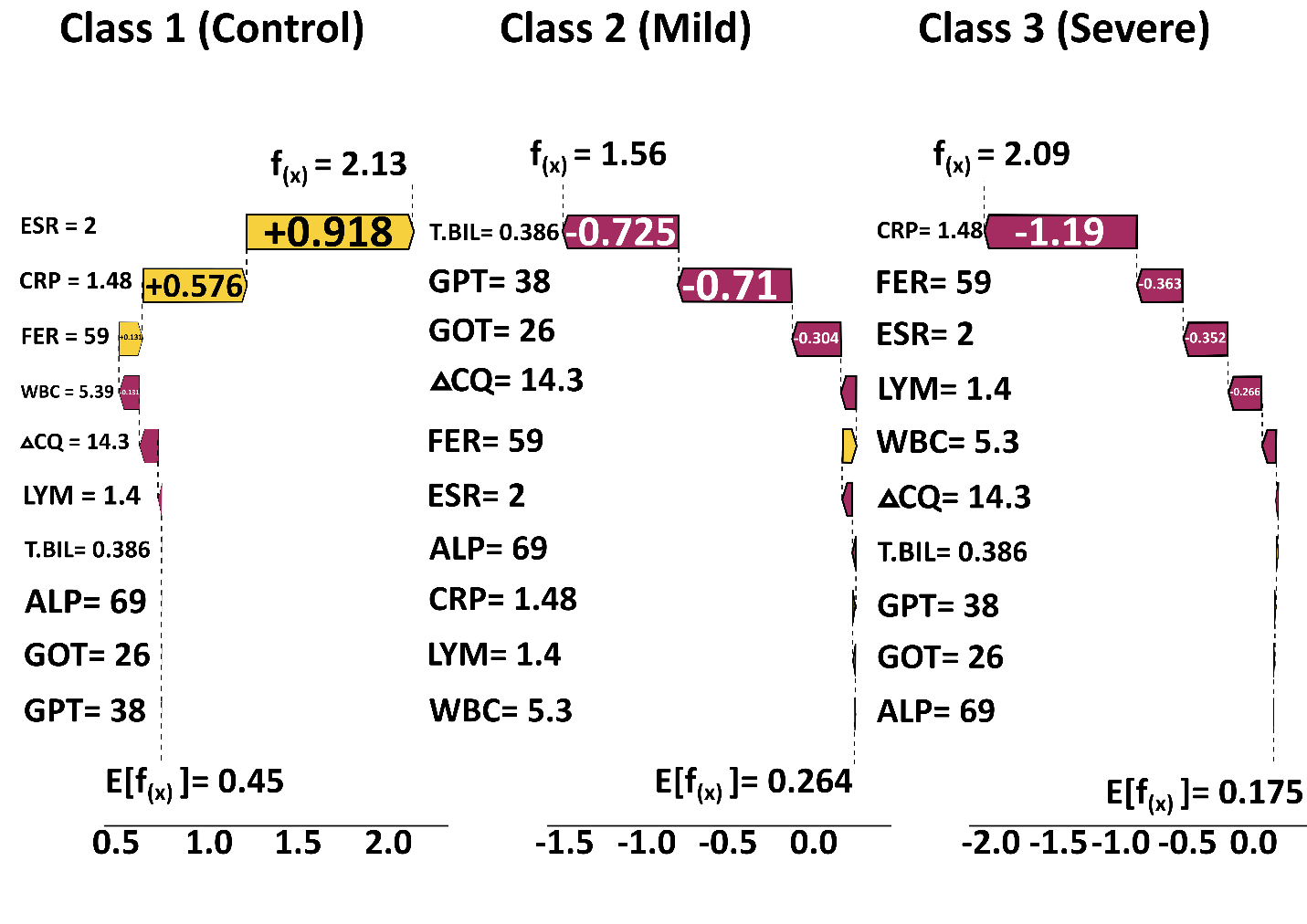


**Figure 9: SHAP Force Plots for Severity Classes.**

According to class-specific force plots, higher CRP and ESR values were linked to an increased contribution toward severe classification in the exploratory model, while elevated GPT and T.BIL showed comparatively higher contributions to mild classification.

1. **List of reagents, instruments, manufacturers, and catalog numbers used in laboratory procedures.**

| Reagents, instruments | Company | Country and Cat. No |
| --- | --- | --- |
| QIAGEN QIAamp RNA Blood Mini Kit | Qiagen | Germany, 52304 |
| β-Mercaptoethanol | ScharLab | Spain |
| Primers | Sigma-Aldrich/Merck KGaA | Germany |
| qPCRBIO SYGreen 1-Step Go Lo-ROX | PCR Biosystems | UK, PB25.31-03 |
| CFX96 Real-Time PCR Detection System | Bio-Rad Laboratories | USA |
| Microcentrifuge | DLAB Scientific | China |
| Water bath | BIOBASE | China |
| NanoDrop spectrophotometer /EzDrop1000 | BLUE-RAY biotech | Taiwan |
